# HER2 testing on core needle biopsy specimens from primary breast cancers: interobserver reproducibility and concordance with surgically resected specimens

**DOI:** 10.1186/1471-2407-10-534

**Published:** 2010-10-07

**Authors:** Hitoshi Tsuda, Masafumi Kurosumi, Shinobu Umemura, Sohei Yamamoto, Takayuki Kobayashi, Robert Yoshiyuki Osamura

**Affiliations:** 1 Diagnostic Pathology Section, Clinical Laboratory Division, National Cancer Center Hospital, 5-1-1 Tsukiji, Chuo-ku, Tokyo 104-0045, Japan; 2 Department of Pathology, Saitama Cancer Center Hospital, 818 Komuro, Ina-machi, Kitaadachi-gun, Saitama 362-0806, Japan; 3 Department of Pathology, Tokai University School of Medicine, Shimokasuya 143, Isehara, Kanagawa 259-1193, Japan; 4 Department of Basic Pathology, National Defense Medical College, 3-2 Namiki, Tokorozawa, Saitama 359-8513, Japan

## Abstract

**Background:**

Accurate evaluation of human epidermal growth factor receptor type-2 (HER2) status based on core needle biopsy (CNB) specimens is mandatory for identification of patients with primary breast cancer who will benefit from primary systemic therapy with trastuzumab. The aim of the present study was to validate the application of HER2 testing with CNB specimens from primary breast cancers in terms of interobserver reproducibility and comparison with surgically resected specimens.

**Methods:**

A total of 100 pairs of archival formalin-fixed paraffin-embedded CNB and surgically resected specimens of invasive breast carcinomas were cut into sections. All 100 paired sections were subjected to HER2 testing by immunohistochemistry (IHC) and 27 paired sections were subjected to that by fluorescence in situ hybridization (FISH), the results being evaluated by three and two observers, respectively. Interobserver agreement levels in terms of judgment and the concordance of consensus scores between CNB samples and the corresponding surgically resected specimens were estimated as the percentage agreement and κ statistic.

**Results:**

In CNB specimens, the percentage interobserver agreement of HER2 scoring by IHC was 76% (κ = 0.71) for 3 × 3 categories (0-1+ *versus *2+ *versus *3+) and 90% (κ = 0.80) for 2 × 2 categories (0-2+ *versus *3+). These levels were close to the corresponding ones for the surgically resected specimens: 80% (κ = 0.77) for 3 × 3 categories and 92% (κ = 0.88) for 2 × 2 categories. Concordance of consensus for HER2 scores determined by IHC between CNB and the corresponding surgical specimens was 87% (κ = 0.77) for 3 × 3 categories, and 94% (κ = 0.83) for 2 × 2 categories. Among the 13 tumors showing discordance in the mean IHC scores between the CNB and surgical specimens, the results of consensus for FISH results were concordant in 11. The rate of successful FISH analysis and the FISH positivity rate in cases with a HER2 IHC score of 2+ differed among specimens processed at different institutions.

**Conclusion:**

It is mandatory to study HER2 on breast cancers, and either CNB or surgical specimen can be used.

## Background

The human epidermal growth factor receptor type-2 (*HER2*) proto-oncogene (c-*erb*B-2) is amplified in 15-30% of human breast cancers, causing overexpression of its protein. *HER2 *gene amplification and/or protein overexpression is an indicator of clinical tumor aggressiveness [1-3]. The efficacy of trastuzumab, a humanized anti-HER2 monoclonal antibody, against breast cancers with *HER2 *gene amplification and/or protein overexpression has been established in clinical trials for patients with metastatic breast cancer or those with operable primary breast cancer as adjuvant systemic therapies [4-7]. Furthermore, as neoadjuvant therapy for patients with breast cancers showing HER2 amplification and/or overexpression, therapies involving a combination of trastuzumab and chemotherapy have been shown to be effective in achieving a complete pathological tumor response [8,9].

HER2 testing comprises immunohistochemistry (IHC) to examine protein overexpression and fluorescence *in situ *hybridization (FISH) to examine gene amplification. These tests are performed on tissue sections of routinely-processed formalin-fixed, paraffin-embedded tumors. High accuracy is required for these HER2 tests in order to identify patients who would benefit from trastuzumab therapy. For the test algorithm, it is generally recommended that IHC is performed first, and that FISH is added if the result of IHC is equivocal [10-12]. Studies of quality assessment have demonstrated that interobserver agreement levels are high for tumors with an IHC score of 0 or 1+, or those with a score of 3+, but that the level is generally low for those with a score of 2+ [13-16]. A higher interobserver agreement level can be achieved with FISH than with IHC, especially for tumors with an IHC score of 2+ [13,14,16]. It has also been shown that the quality of both tests is higher at institutions that perform a larger number of HER2 tests than at those where a smaller number of such tests are performed [17-19].

In recent years, core needle biopsies (CNBs) have been used for the qualitative diagnosis of breast tumors. Because of the prevalence of primary systemic therapies as a standard treatment for primary breast cancers, not only hormone receptor status but also HER2 status are generally assayed from CNB specimens to test the eligibility of patients for primary systemic therapy with trastuzumab [20]. However, it would be expected that examination of CNB specimens alone might result in a proportion of false-positive and/or false-negative results, because CNB samples represent only part of the tumor, notwithstanding the possible presence of intratumor heterogeneity [21-25]. Furthermore, because of the small volume of CNB specimens, the interobserver agreement rate of HER2 tests for CNB specimens might be lower than for surgically resected specimens.

Therefore, we examined the levels of interobserver agreement for HER2 status determination in both CNB specimens and corresponding surgically resected specimens from 100 patients with primary breast cancer who had not received primary systemic therapy. We compared the HER2 protein status determined by IHC between the CNB specimens and surgically resected specimens of the same tumor. We also compared the HER2 protein status determined by IHC, with the *HER2 *gene status determined by FISH, in CNB specimens and in the corresponding surgically resected specimens. On the basis of these results, we evaluated the utility and challenges of HER2 testing within CNB specimens.

## Methods

### Tissue samples

We examined 100 paired samples of invasive breast carcinoma obtained by CNB and surgical resection from patients treated at Saitama Cancer Center, Ina, Saitama (50 cases), Tokai University Hospital, Isehara, Kanagawa (25 cases), and the National Defense Medical College Hospital, Tokorozawa, Saitama (25 cases), Japan. At all three institutions, HER2 testing is performed very frequently for routine diagnostics and/or for studies of quality assessment. Collaborating pathologists in the three institutes were assigned to submit almost equal number of CNB cases of each score (score 0 or 1+, 2+, and 3+), for the purpose that almost equal number of HER2-negative, equivocal and positive cases were examined in the study. However, these institutional scores were not informed to the pathologists on the central review. Therefore, the cases were not consecutive and there was some selection bias.

None of the patients with these tumors had received neoadjuvant therapy before CNB and surgical resection. At each institution, 4-μm-thick sections cut from routinely processed formalin-fixed, paraffin-embedded tissue blocks were subjected to immunohistochemistry and FISH. IHC and FISH assays were performed on parallel slides of the same CNB/surgically resected specimens. The present study was conducted with approval from the internal review board for ethical issues of the National Defense Medical College. Informed consent had been acquired from each patient for the purpose of general research use of surgically resected tissues, and the requirement for informed consent for the present study was waived by the internal review board according to the guideline of ethical issues for epidemiologic studies by the Ministry of Health, Labor and Welfare and the Ministry of Education, Culture, Sports, Science and Technology, Japan.

### Immunohistochemistry

Expression of HER2 oncoprotein was examined using HercepTest II (Dako, Glostrup, Denmark) in the Dako Japan Central Laboratory, Tokyo, Japan. Deparaffinization, antigen retrieval, and immunohistochemistry were performed to 100 CNB and 100 paired surgically resected tumor sections in accordance with the manufacturer's instructions using an Autostainer Plus (Dako) [16].

Three experienced (for >25 years) pathologists (M.K., S.U., H.T.), being blinded from institutional IHC results or the present FISH results, independently evaluated the results of IHC, and assigned a score of 0 (no staining), 1+ (weak, incomplete membrane staining in any proportion of tumor cells), 2+ (complete membrane staining that is either nonuniform or weak in intensity but with obvious circumferential distribution in at least 10% of tumor cells, or invasive tumors show intense, complete membrane staining of 30% or fewer tumor cells), or 3+ (uniform, intense membrane staining of >30% of invasive tumor cells) in accordance with the guidelines of the American Society of Clinical Oncology (ASCO)/College of American Pathologists (CAP) [10]. Scores of 0 and 1+ were categorized as IHC-negative, a score of 2+ was categorized as equivocal, and a score of 3+ was categorized as overexpression (positive). If the score assigned by three observers differed among the three categories, the majority scores were acquired as consensus judgments. When the judgments of the three observers differed from each other, the median value was acquired as the representative score.

### FISH

FISH was performed on all 100 CNB specimens and on 27 surgically resected specimens for which the consensus judgment of the IHC result differed from that of the CNB result and/or was discordant among the three observers.

FISH was performed manually using a PathVysion *HER*-2 DNA probe kit (Abbott Molecular, Wiesbaden, Germany) in accordance with the manufacturer's instructions [13] at the Department of Basic Pathology, National Defense Medical College, Tokorozawa, Saitama, Japan. The slides were visualized using a Leica DMR fluorescence microscope (Leica, Cambridge, UK). Two observers (S.Y. or T.K. and H.T.), being blinded from IHC results and FISH results of the corresponding CNB or surgical resection specimen, counted the signals for *HER2 *and *CEP17 *on a total of 40 cancer cell nuclei. The total number of *HER2 *signals was divided by the total number of *CEP17 *signals on 40 nuclei, and the *HER2*/*CEP17 *ratio was calculated. *HER2 *gene amplification was judged as positive, equivocal, and negative if the *HER2*/*CEP17 *ratio was more than 2.2, 1.8 to 2.2, and less than 1.8, respectively [10]. When a tumor was judged as equivocal, 40 additional nuclei in another tumor area on invasion were counted again by two observers. If the *HER2*/*CEP17 *ratio was 2.0 or higher in the re-test, the tumor was finally judged as having *HER2 *amplification.

### Interobserver agreement

With regard to IHC and FISH, interobserver agreement of judgments and the concordance of consensus scores between the CNB and corresponding surgically resected specimens were estimated in terms of percentage agreement and the κ statistic. The percentage of agreement was calculated as follows:

(Number of tumors to which the three observers assigned an identical score/total number of tumors) × 100

The level of agreement was categorized as almost perfect, substantial, moderate, fair, and slight when the κ value was >0.80, >0.60-0.80, >0.40-0.60, >0.20-0.40, and 0-0.20, respectively [26,27].

## Results

### Interobserver agreement for IHC and FISH

For CNB specimens, the percentage interobserver agreement for HER2 scores determined by IHC was 76% for 3 × 3 categories (0-1+ vs 2+ vs 3+) and 90% for 2 × 2 categories (0, 1+ or 2+ vs 3+). In terms of the κ statistic, the interobserver agreement levels were substantial for the 3 × 3 categories (κ = 0.71, σ = 0.065) and the 2 × 2 categories (κ = 0.80, σ = 0.075). The 24 tumors for which judgment discordance arose for 3 × 3 categories are shown in Table 1. All of these tumors showed discordance in one score, and none showed a difference in 2 scores.

**Table 1:** Core needle biopsy tumor specimens for which interobserver disagreement arose regarding the results of HER2 immunohistochemistry

Code	Final score	IHC score			FISH *(HER2/CEP17)*
			
		Observer A	Observer B	Observer C	
B45	1+	1+	1+	2+	- (0.96)
B78	1+	1+	1+	2+	- (0.99)
B24	1+	2+	1+	1+	- (1.01)
B82	1+	2+	1+	1+	- (1.02)
B79	1+	2+	1+	1+	- (1.05)
B99	1+	1+	1+	2+	- (1.07)
B36	1+	1+	1+	2+	- (1.11)
B22	2+	2+	1+	2+	- (0.97)
B69	2+	2+	1+	2+	- (1.04)
B43	2+	2+	1+	2+	- (1.09)
B86	2+	2+	1+	2+	- (1.39)
B29	2+	2+	2+	1+	- (1.42)
B100	2+	2+	1+	2+	+ (2.38)
B16	2+	3+	2+	2+	+ (2.56)
B97	2+	2+	1+	2+	+ (4.56)
B52	2+	3+	2+	2+	+ (5.44)
B91	2+	2+	2+	3+	+ (6.80)
B90	2+	3+	2+	2+	+ (10.38)
B102	2+	3+	2+	2+	+ (12.62)
B50	3+	3+	3+	2+	+ (5.11)
B7	3+	3+	2+	3+	+ (8.83)
B96	3+	3+	2+	3+	+ (9.02)
B95	3+	3+	2+	3+	+ (10.92)
B27	3+	3+	3+	2+	+ (12.50)

The HER2 score was assigned according to the 2007 ASCO/CAP guideline [10]. The majority score was assigned as the final score for the tumor.

FISH, fluorescence in situ hybridization; IHC, immunohistochemistry

For surgically resected specimens, the percentage interobserver agreement for HER2 scores determined by IHC was 80% for the 3 × 3 categories and 92% for the 2 × 2 categories. In terms of the κ statistic, the interobserver agreement level was substantial for the 3 × 3 categories (κ = 0.77, σ = 0.060) and almost perfect for the 2 × 2 categories (κ = 0.88, σ = 0.051). The 20 tumors for which judgment discordance arose for 3 × 3 categories are shown in Table 2. All of these tumors showed discordance in one score, and none showed a difference in 2 scores.

**Table 2:** Surgically resected tumor specimens for which interobserver disagreement arose regarding the results of HER2 immunohistochemistry.

Code	Final score	IHC score			FISH (*HER2/CEP17*)
			
		Observer A	Observer B	Observer C	
S54	0+	0	2+	0	- (0.92)
S17	1+	1+	1+	2+	NA
S26	1+	1+	1+	2+	- (0.68)
S78	1+	1+	1+	2+	- (0.82)
S79	1+	2+	1+	1+	- (0.89)
S76	1+	2+	1+	1+	- (0.95)
S82	1+	2+	1+	1+	- (0.97)
S77	1+	2+	1+	1+	- (1.47)
S23	2+	2+	1+	2+	- (0.47)
S22	2+	2+	1+	2+	- (0.92)
S16	2+	3+	2+	2+	- (1.03)
S84	2+	2+	1+	2+	- (1.13)
S67	2+	2+	1+	2+	+ (2.24)
S97	2+	3+	2+	2+	+ (3.89)
S103	2+	3+	2+	2+	+ (7.75)
S91	2+	3+	2+	2+	+ (7.77)
S94	3+	3+	3+	2+	+ (3.61)
S52	3+	3+	3+	2+	+ (5.09)
S42	3+	3+	3+	2+	+ (9.24)
S18	3+	3+	3+	2+	+ (12.40)

The HER2 score was assigned according to the 2007 ASCO/CAP guideline [10]. The majority score was assigned as the final score for the tumor. NA, not available because FISH was not successful.

FISH, fluorescence in situ hybridization; IHC, immunohistochemistry

The consensus score for HER2 expression determined by IHC was 0 or 1+, 2+, and 3+ in 60, 19, and 21 tumors, respectively, in CNB specimens, and in 60, 15, and 25 tumors, respectively, in surgically resected specimens.

FISH was successful in 99 of the 100 CNB specimens. By the first examinations, 96 tumors (97%) were judged to have the same score by two observers. Because three other tumors were judged differently by two observers and the average of the two judgments was within the range of equivocal, they were subjected to a re-count: for two tumors, the second judgments also differed between two observers, being positive and equivocal respectively, but the average of the judgments of *HER2*/*CEP17 *ratio exceeded 2.20, so they were finally judged positive. For the other, the second judgments were commonly positive (Table 3). By FISH, *HER2 *gene amplification was finally judged positive in 33 CNB specimens (33%) but negative in 66 (67%).

**Table 3:** Tumor specimens for which interobserver disagreement arose regarding the results of fluorescence in situ hybridization.

CNB specimens							
**Code**	**Final judgment**	***HER2/CEP17 *by 1st counts**			***HER2/CEP17 *by 2nd counts**		
			
		**Obs. A**	**Obs. B**	**Average**	**Obs. A**	**Obs. B**	**Average**

B61	Amplification	2.71	1.55	2.13	2.55	1.88	2.22
B62	Amplification	2.35	1.88	2.12	2.44	2.07	2.26
B87	Amplification	1.94	2.44	2.19	3.50	2.38	2.44

Surgically resected specimens							

Code	Final judgment	*HER2/CEP17 *by 1st counts			*HER2/CEP17 *by 2nd counts		
			
		Obs. A	Obs. B	Average	Obs. A	Obs. B	Average

S67	Amplification	2.67	1.81	2.24	ND	ND	-

HER2 amplification was defined as positive, equivocal, and negative when the *HER2/CEP17 *ratio was more than 2.2, between 1.8 and 2.2, and less than 1.8, respectively. For the surgically resected specimen, the average of the *HER2/CEP17 *ratio (2.24) between observers (2.67 and 1.82) was adopted, because the second count was not done (ND). Obs., observer

A total of 27 surgically resected tumors were subjected to FISH. Among the 25 surgical specimens for which FISH was successful, only one (no. 67) was judged differently by two observers, being positive and equivocal respectively. However, the average of the two judgments was within the range of positive (*HER2*/*CEP17 *ratio = 2.236), the case was judged as positive without re-evaluation (Table 3). In total, *HER2 *gene amplification in surgically resected specimens was positive in 12 (48%) and negative in 13 (52%).

FISH was not successful in three specimens: one CNB and two surgically resected specimens. All three of these specimens were processed in institution A.

### Comparison of IHC and FISH test results between CNB and surgically resected specimens

**Figure 1:**
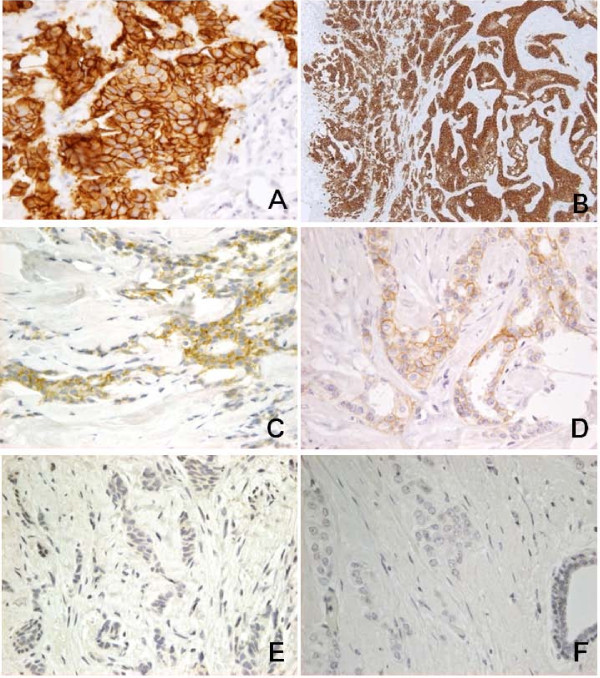
**Cases with concordant judgment of HER2 score between CNB and surgically resected specimens**. A-B. Case 3: HER2 score for both the CNB specimen (A) and the surgically resected specimen (B) was 3+. C-D. Case 22: HER2 score for both the CNB specimen (C) and the surgically resected specimen (D) was 2+. E-F. Case 2: HER2 score for both the CNB specimen (E) and the surgically resected specimen (F) was 0. Immunoperoxidase reaction, original magnification ×200.

Concordance in consensus HER2 IHC scores between CNB and the corresponding surgically resected specimens was 87% for 3 × 3 categories, and 94% for 2 × 2 categories (Table 4). The κ statistic indicated that their concordance was substantial for the 3 × 3 categories (κ = 0.77, σ = 0.045) and almost perfect for the 2 × 2 categories (κ = 0.83, σ = 0.038). Representative concordant cases are presented in Figure 1.

**Table 4:** Concordance of consensus HER2 judgments by immunohistochemistry between core needle biopsy and corresponding surgically resected specimens.

3 categories (0 or 1+ vs. 2+ vs. 3+)			
	
HER2 score for surgically resected specimens	Number of tumors		
	
	HER2 score for CNB specimens		
	
	0 or 1+	2+	3+
0 or 1+	56	3	1
2+	4	11	0
3+	0	5	20

% agreement = 87%, κ = 0.77, standard deviation (σ) = 0.045			

2 categories (0, 1+. or 2+ vs. 3+)			

HER2 score for surgically resected specimens	Number of tumors		
	
	HER2 score for CNB specimens		
	
	0, 1+ or 2+		3+

0, 1+, or 2+	74		1
3+	5		20
% agreement = 94%, κ = 0.83, σ = 0.038			

The judgments were performed according to the 2007 ASCO/CAP guideline.

Among the 60 tumors with a score of 0 or 1+ for CNB specimens, 56 (93%) were also judged to have a score of 0 or 1+ but the other four were scored 2+ for the surgically resected specimens. Among the 21 tumors with a score of 3+ for CNB specimens, 20 (95%) were also judged to have a score of 3+ and only one (5%) was scored 0 for the surgically resected specimens. In contrast, among the 19 tumors with a score of 2+ for CNB specimens, only 11 (58%) were also scored 2+ for the surgically resected specimens. In three and five of these 19 cases, scores of 0 or 1+ and 3+ were assigned, respectively, for the surgically resected specimens.

Overall, consensus HER2 IHC scores showed discordance between CNB and surgically resected specimens in 13 of 100 cases (Table 5). The discordance may have been attributable to intratumor heterogeneity, suboptimal processing of the specimens, and/or the borderline nature of the tumor. The borderline nature means that the state of HER2 expression was borderline between 1+ and 2+ or between 2+ and 3+, namely, the conditions that it was difficult to judge whether the entirely circumscribing membrane immunoreactivity of the HER2 was moderate (2+) or strong (3+), or whether the weak membrane HER2 immunoreactivity was entirely (2+) or incompletely (1+) circumscribing the membrane.

**Table 5:** 13 tumors for which interobserver disagreement arose regarding the results of immunohistochemistry.

Code No.	Immunohistochemistry			FISH	
		
--	CNB	Surgery	Interpretation	CNB	Surgery
84	0 (0/0/0)	2+(2/1/2)	Processing, hetero	Neg	Neg
23	1+(1/1/1)	2+(2/1/2)	Heterogeneity	Neg	Neg
24	1+(2/1/1)	2+(2/2/2)	Heterogeneity	Neg	NA
67	1+(1/0/1)	2+(2/1/2)	Processing, border	Neg	Pos
10	2+(2/2/2)	1+(1/1/1)	Processing	Neg	Neg
43	2+(2/1/2)	0 (0/0/0)	Processing	Neg	Neg
69	2+(2/1/2)	1+(1/1/1)	Processing, border	Neg	Neg
52	2+(3/2/2)	3+(3/3/2)	Processing, border	Pos	Pos
90	2+(3/2/2)	3+(3/3/3)	Processing, border	Pos	Pos
92	2+(2/2/2)	3+(3/3/3)	Predominantly DCIS	Pos	Pos
94	2+(2/2/2)	3+(3/3/2)	Processing, border	Pos	Pos
102	2+(3/2/2)	3+(3/3/3)	Processing, border	Pos	Pos
54	3+(3/3/3)	0 (0/2/0)	Heterogeneity	Neg	Neg

FISH, fluorescence in situ hybridization; NA, Data were not available; neg, negative; pos, positive; parenthesis, judgments of scores by three observers (Underlines indicate interobserver disagreement.)

**Figure 2:**
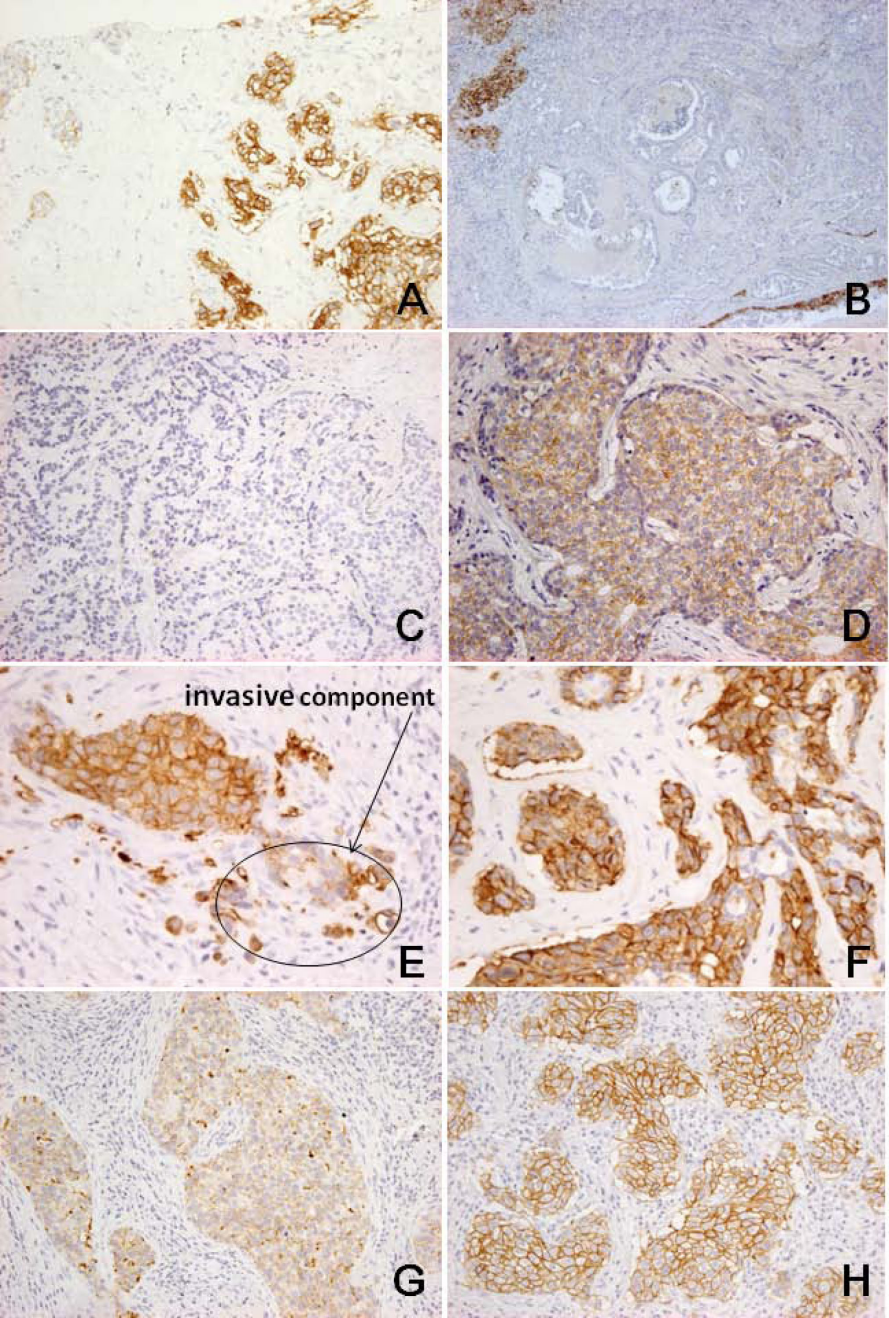
**Cases with discordant judgment of HER2 score between CNB and surgically resected specimens**. A-B. Case 54: HER2 score was 3+ for the CNB specimen (A) but 0 for the surgically resected specimen (B). The tumor had heterogeneous HER2 expression, and >30% of the area showed a strong membrane immunoreaction in the CNB specimen, whereas most of the area in the surgically resected specimen was HER2-negative. C-D. Case 84: HER2 score was 0 for the CNB specimen (C) but 2+ for the surgically resected specimen (D). This difference might have been due to suboptimal processing of CNB specimens or intratumor heterogeneity. E-F. Case 92: HER2 score was 2+ for the CNB specimen (E) but 3+ for the surgically resected specimen (F). Because the CNB specimen contained only a small amount of invasive component, the evaluation of HER2 was difficult. G-H. Case 94: HER2 score was 2+ for the CNB specimen (G) but 3+ for the surgically resected specimen (H). Processing of the CNB specimen might have been suboptimal, or the tumor may have been borderline in nature. Immunoperoxidase reaction, original magnification ×200.

In at least five tumors (Nos. 23, 24, 54, 84, 92), the discordance appeared to have been due to intratumor heterogeneity. In case 54, to the surgically resected specimen, all three observers gave a score of 3+ because >30% of cancer cells showed strong membrane staining. In contrast, in the CNB specimen, the percentage of cancer cells showing strong membrane staining was around 10%, and two observers gave a score of 0, and the other gave one of 2+ (Figure 2A). In cases 23, 24, and 84, there appeared to be intratumor heterogeneity ranging between areas of moderate, and no or weak, HER2 staining (2+ vs 0 or 1+) (Figure 2B). In case 92, the HER2 score for the predominant intraductal component was uniformly 3+, but in the focus of invasive carcinoma, the immunoreaction was weaker (Figure 2C). In this case, we performed FISH on the part including both invasive carcinoma and non-invasive components.

In 4 cases (Nos. 52, 90, 94 and 102), the HER2 score was 2+ for the CNB specimen but 3+ for the surgical specimen (Figure 2D). In these cases, the membrane HER2 immunoreaction in CNB specimens was weaker and less continuous than that in surgically resected specimens. In 5 other cases (Nos. 10, 43, 52, 67 and 69), the intensity of the HER2 immunoreaction differed between the CNB and surgically resected specimens, but the immunoreaction pattern in the surgically resected specimens was uniform. Therefore, suboptimal processing of CNB specimens or long-term fixation of surgically resected specimens might have been partly responsible for the difference.

Interobserver disagreement regarding the HER2 score arose for either CNB specimens or surgically resected specimens in 11 of these 13 cases. Possible reasons for this may have been the borderline nature of the tumors, preanalytical factors, and/or inratumor heterogeneity (e.g., No. 54) (Table 5).

In 23 (92%) of the 25 tumors for which HER2 FISH was performed, consensus judgments regarding *HER2 *gene status were concordant between the CNB and the surgically resected specimens, being amplified in 11 and not amplified in 12 (Table 6). In the other two cases, the consensus judgments were discordant between the CNB and the surgically resected specimens: In one (no. 67), the CNB specimen showed an IHC score of 1+ with a *HER2/CEP17 *ratio of 0.84, whereas the surgically resected specimen showed an IHC score of 2+ with a *HER2/CEP17 *ratio of 2.24. As mentioned above, the case was judged differently by two observers, but the average of the judgments made a score of positive (Table 3). In the other, both the CNB and surgically resected specimens showed an IHC score of 2+, and the *HER2/CEP17 *ratio was 1.03 and 2.56, respectively (No. 16). For that case, there was no disagreement in HER2 score of CNB and surgically resected specimens by IHC.

**Table 6:** Concordance of consensus HER2 FISH results of between core needle biopsy and surgically resected specimens.

*HER2/CEP17 *ratio for surgically resected specimens	Number of tumors		
	
	*HER2/CEP17 *ratio for CNB specimens		
	
	> 2.2	1.8 to 2.2	<1.8
Higher than 2.2	11	0	1
1.8 to 2.2	0	0	0
<1.8	1	0	12

% agreement = 92%			

The judgments were performed according to the 2007 ASCO/CAP guideline. For two tumors, data on CNB and/or surgically resected specimens were not available.

### Correlation between IHC and FISH results

Among the 100 CNB specimens, the percentage of *HER2 *gene amplification was 95% (20 of 21), 58% (11 of 19), and 3% (2 of 60) for HER2 IHC scores of 3+, 2+, and 0 or 1+, respectively (Table 7). With regard to tumors with an IHC score of 2+, the ratio of *HER2 *gene amplification differed between tumor specimens from different institutions. In tumor specimens from institutions A and B, the rates of *HER2 *gene amplification were 20% (1 of 5) and 33% (1 of 3), respectively, but in specimens from institute C, the rate was as high as 82% (9 of 11).

**Table 7:** Correlation between consensus immunohistochemistry and FISH results for core needle biopsy and surgically resected specimens.

Core needle biopsy specimens				
	**Number of tumors (%)**			
	
	**Total**	***HER2 *gene amplification by FISH**		
		
		**Positive**	Equivocal*	**Negative**

HER2 IHC score				
3+	21	20 (95)	0 (0)	1 (5)
2+	19	11 (58)	0 (0)	8 (42)
0 or 1+	60	2 (3)	0 (0)	58 (97)

Total	100	33 (33)	0 (0)	67 (67)

Surgically resected specimens				

	Number of tumors (%)			
	
	Total	*HER2 *gene amplification by FISH		
		
		Positive	Equivocal*	Negative

HER2 IHC score				
3+	10	10(100)	0 (0)	0 (0)
2+	7	3 (43)	0 (0)	4 (57)
0 or 1+	8	0 (0)	0 (0)	8 (100)

Total	25	13 (52)	0 (0)	12 (48)

*For three tumors, FISH judgment was equivocal by the first counts, but by the second counts, the judgments changed into positive (Table 3). FISH, fluorescence in situ hybridization

For the 25 surgically resected specimens successfully subjected to FISH, the percentage of *HER2 *gene amplification was 100% (10 of 10), 43% (3 of 7), and 0% (0 of 8) for HER2 IHC scores of 3+, 2+, and 0 or 1+, respectively (Table 7). In tumor specimens from institution A, the rate of *HER2 *gene amplification in IHC 2+ cases was 0% (0 of 3), whereas in specimens from institution C, the rate was 80% (4 of 5). No cases with a score of 2+ were subjected to FISH at institution B.

For CNB specimens, 14 cases showed interobserver disagreement in HER2 IHC scores of between 1+ and 2+, and, of these, seven tumors each were finally scored as 1+ and 2+. Among these tumors, *HER2 *gene amplification was detected in 0 (0%) and 2 (29%), respectively. The other 10 cases showed interobserver disagreement in HER2 IHC scores of between 2+ and 3+, and, of these, five tumors each were finally scored as 2+ and 3+. *HER2 *gene amplification was detected in all of these tumors (Table 1).

For surgically resected specimens, 12 cases showed interobserver disagreement between IHC scores of 0/1+ and 2+, and eight and four tumors were finally scored as 0/1+ and 2+, respectively. Among these tumors, *HER2 *gene amplification was detected in 0 (0%) and 1 (25%), respectively. The other eight showed interobserver discordance between IHC scores of 2+ and 3+, and four tumors each were finally scored as 2+ and 3+. Among these tumors, *HER2 *gene amplification was detected in 3 (75%) and 4 (100%), respectively (Table 2).

In the 12 tumors showing discordance of the IHC score for HER2 expression between the CNB and surgically resected specimens, and for which the FISH assay was successful, judgments for the FISH results were concordant in 11 (92%) (Table 5).

## Discussion

From the viewpoint of interobserver agreement level, the percentage agreement levels and κ values among the three observers with regard to the IHC test results were similar between the data for CNB specimens and those for surgically resected specimens. Furthermore, the present study also clarified that the results of IHC for HER2 were mostly concordant between the CNB specimen and the surgically resected specimen from an identical invasive breast cancer. These results indicated that the CNB specimens were of adequate quality for the evaluation of HER2 status by IHC, and that the IHC scores obtained for the CNB specimens were mostly representative of the HER2 IHC scores for the entire tumor specimen. Therefore, HER2 testing using IHC for CNB specimens appeared to be valid for a majority of primary breast cancers. The present results were similar to the very first studies done by Chivukula et al. that stated as CNB a better sample [25].

Previous reports have indicated that concordance of HER2 IHC scores between CNB and corresponding excisional biopsy/surgically resected specimens was 87-98.8% [21-23,28]. The introduction of FISH analysis has improved the concordance rate for HER2 status [22,25]. In the study by Apple et al., the concordance rate of 87% for IHC was improved to 92% by FISH [22]. Intratumor heterogeneity for HER2 amplification was reported to be present in 13% of tumors of an IHC score of 3+, and was especially higher in those with low-grade amplification (ratio >2.2 to <4.0) [24]. For tumors with an IHC score of 2+, the incidence of intratumor heterogeneity in HER2 scores was also relatively frequent (14%), but FISH analysis of CNB specimens almost completely resolved the issue of heterogeneous HER2 expression [25].

Some studies had suggested that the validity of IHC score 3+ in core biopsies was limited, reporting high rates of false positives (19.3%). However, Moelans et al, showed that there was only a slightly higher percentage of IHC 3+ positivity in biopsies compared to resections, and that did not reach statistical difference [29]. Their results are in line with those in the present study (25% vs 21%). In the present study, it was confirmed that concordance of the HER2 test results was higher for FISH (98%) than for IHC (87%). Among the 13 tumors that showed discordance of HER2 IHC scores between the CNB and surgically resected specimens, 11 showed concordance of the results obtained by FISH.

The disagreement in the results obtained with IHC between CNB and surgical specimens appeared to be derived from 1. intratumor heterogeneity, 2. pre-analytical factors including variations in the duration of fixation and suboptimal tissue processing, and/or 3. borderline tumor properties. To overcome the problem of intratumor heterogeneity in HER2 expression, examination of a large volume of tumor tissue appears to be necessary. To solve the problem of borderline tumor properties in terms of HER2 expression, the introduction of judgment by multiple observers and/or DNA copy analyses might be of value.

From the present results, the rate of *HER2 *gene amplification appeared to be low when interobserver discordance was seen between IHC scores of 0/1+ and 2+, whereas most tumors had gene amplification when there was interobserver discordance between scores of 2+ and 3+. These findings will be helpful for the decision if subsequent FISH should be performed or not. For 2+/3+ discrepancies FISH test should always be added because the percentage of HER2 amplification is high. Counting of *HER2 *gene copies by FISH, chromogenic in situ hybridization (CISH), silver-enhanced in situ hybridization (SISH), or dual-color dual-hapten in situ hybridization (DDISH) would greatly improve the concordance of HER2 status between CNB and surgically resected specimens [30-32].

Another important factor to be considered is the preanalytical condition of the specimens. In the present study, FISH analysis was sometimes unsuccessful for specimens processed at institution A, whereas FISH was always successful for specimens processed at institutions B and C. Interview revealed that the suboptimal FISH results obtained at institution A were attributable to long-term (about 1 week) fixation of the specimens. It was shown that a prolonged formalin fixation could lose FISH amplification and/or yield to unsuccessful test (Hiroi S, Tsuda H et al, manuscript in preparation) [33].

On the other hand, the localization of the HER2 immunoreaction on the cancer cell membrane was not uniform in CNB specimens from HER2-positive tumors processed at institution C, whereas this was not an evident feature in specimens processed at other institutions or in surgically resected specimens. The reason for this unusual immunoreaction is unclear. It has been shown that quality assessment of HER2 tests is very important for identifying patients who are very likely to benefit from therapy with trastuzumab [10,34,35]. For quality assessment, not only improvement of the interobserver agreement level but also standardization of pre-analytical specimen preparation should be taken into consideration.

## Conclusion

We have clarified that CNB specimens showed almost equal reliability to surgically resected specimens for testing of HER2 expression in terms of interobserver agreement levels and concordance with FISH results. In most of specimens with equivocal IHC results, accurate HER2 status was known determined by retesting with FISH. To further improve the reliability of HER2 tests using CNB specimens, it might be useful to sample a larger volume of tumor tissue, to conduct evaluation by multiple observers, and to take measures to improve the pre-analytical conditions of the specimens.

## List of abbreviations used

CNB: core needle biopsy; FISH: fluorescence in situ hybridization; HER2: human epidermal growth factor receptor type-2; IHC: immunohistochemistry

## Competing interests

The authors declare that they have no competing interests.

## Authors' contributions

HT conceived the study, acquired data of IHC and FISH, analyzed data, and prepared manuscript. MK and SU also conceived the study, acquired data of IHC and analyzed data. SY and TK acquired data of FISH and analyzed data. RYO also conceived the study and supervised the analysis. All authors read and approved the final manuscript.

## Pre-publication history

The pre-publication history for this paper can be accessed here:

http://www.biomedcentral.com/1471-2407/10/534/prepub
